# Microfibrillated Cellulose with a Lower Degree of Polymerization; Synthesis via Sulfuric Acid Hydrolysis under Ultrasonic Treatment

**DOI:** 10.3390/polym15040904

**Published:** 2023-02-11

**Authors:** Yuriy N. Malyar, Irina G. Sudakova, Valentina S. Borovkova, Anna I. Chudina, Elena V. Mazurova, Sergey A. Vorobyev, Olga Yu. Fetisova, Eugene V. Elsufiev, Ivan P. Ivanov

**Affiliations:** 1 Institute of Chemistry and Chemical Technology, Krasnoyarsk Science Center, Siberian Branch Russian Academy of Sciences, Akademgorodok 50/24, Krasnoyarsk 660036, Russia; 2 School of Non-Ferrous Metals and Material Science, Siberian Federal University, pr. Svobodny 79, Krasnoyarsk 660041, Russia

**Keywords:** spruce wood, microcrystalline cellulose, sulfuric acid hydrolysis, ultrasonic treatment, microfibrillated cellulose

## Abstract

A new approach is being considered for obtaining microfibrillated cellulose with a low degree of polymerization by sulfuric acid hydrolysis with simultaneous ultrasonic treatment under mild conditions (temperature 25 °C, 80% power control). Samples of initial cellulose, MCC, and MFC were characterized by FTIR, XRF, SEM, DLS, and TGA. It was found that a high yield of MFC (86.4 wt.%) and a low SP (94) are observed during hydrolysis with ultrasonic treatment for 90 min. It was shown that the resulting microfibrillated cellulose retains the structure of cellulose I and has an IC of 0.74. It was found that MFC particles are a network of fibrils with an average size of 91.2 nm. ζ-potential of an aqueous suspension of MFC equal to −23.3 mV indicates its high stability. It is noted that MFC has high thermal stability, the maximum decomposition temperature is 333.9 °C. Simultaneous hydrolysis process with ultrasonic treatment to isolate MFC from cellulose obtained by oxidative delignification of spruce wood allows to reduce the number of stages, reduce energy costs, and expand the scope.

## 1. Introduction

Cellulose is one of the most wide-spread natural polymers, promising as a chemical raw material due to its availability, low cost, and non-toxicity [1].

Cellulose is currently used as a green biopolymer in various branches of manufacture, an alternative to petroleum-based synthetic polymers in the production of biocomposites, in the paper industry, and as a filler in food production and pharmaceuticals [2,3].

A raw for the cellulose production are various lignocellulosic materials, including wood. The large-tonnage industrial waste, (chips and sawdust) formed during wood harvesting and processing is a stable source of raw materials for manufacturing cellulose products [4].

Cellulose is included in woody biomass in the form of a cellulose-hemicellulose-lignin complex linked by chemical and physical bonds. Therefore, wood must be pre-treated to remove lignin and hemicelluloses, which can act as protective layers for cellulose fibrils and create obstacles for their extraction [5]. At present, the catalytic oxidative delignification of woody biomass in organic acids solutions is being intensively developed. Thus, the authors of [6] developed a single-stage method for production of cellulose with a lignin content below 1 wt.% from wood of different types in an acetic acid–hydrogen peroxide–water medium over the TiO_2_ catalyst using the combination of delignification, bleaching, and acid treatment.

The growing interest in the production of the cellulose-based materials with a low polymerization degree is due to their high application potential [7,8]. The micro- and nanocellulose materials exhibit the unique valuable properties; a low density, high stress–strain performances and crystallinity, low thermal expansion, and a large specific surface. They contain hydroxyl groups (-OH), which can be easily modified by chemical methods [9,10].

It is well known that natural cellulose consists mainly of crystalline (ordered) areas interlinked via amorphous (disordered) spacers. The most efficient method for obtaining cellulosic materials with a low degree of polymerization (DP) is the acidic hydrolysis [11,12]. During the occurrence of acid hydrolysis process under controlled conditions, the amorphous (disordered) cellulose areas are hydrolyzed, whereas the crystalline ones remain invariable. As a result, long cellulose fibers are converted into a mixture of shorter insoluble microcrystalline cellulose (MCC) particles. In the acidic hydrolysis, which proceeds under mild conditions, mineral acids are mainly used, especially inexpensive and highly efficient sulfuric and hydrochloric acids [13]. To obtain MCC, cellulose is hydrolyzed to achieve a DP of 100–400, which corresponds to the average degree of polymerization of commercial MCC [14].

Recently, nanofibrillated celluloses with a DP of lower than 100 have been of great interest, since their unique characteristics are attractive for application in medicine, catalysis, textile industry, surface coating, drug delivery, food packaging, etc. [15,16,17,18].

Microfibrillated cellulose (MFC) is produced by mechanical fibrillation, in particular, high pressure homogenization, microfluidization, milling of natural cellulose to nanofibrils, and its ultrasonic treatment [19]. However, the use of mechanical fibers in the MFC production is highly energy consuming. The energy consumption during mechanical defibrillation can be reduced using the pre-treatment methods by enzymatic hydrolysis [20], TEMPO-mediated oxidation [21] or carboxymethylation [22]. To decrease the cellulose loss and enhance the nanocellulose production efficiency, it was proposed to combine the MFC synthesis by the acid hydrolysis and the same combined with the ultrasonic treatment under mild conditions and to determine the composition and physicochemical properties of the products.

The aim of this study was to examine the possibility of reducing the DP of cellulose obtained by oxidative delignification of spruce wood using acid hydrolysis and the same combined with the ultrasonic treatment under mild conditions and to determine the composition and physicochemical properties of the products.

## 2. Materials and Methods

### 2.1. Raw Material

The raw material used was a cellulose product obtained by oxidative delignification of spruce wood using the method developed in [23].

**Figure 1:**
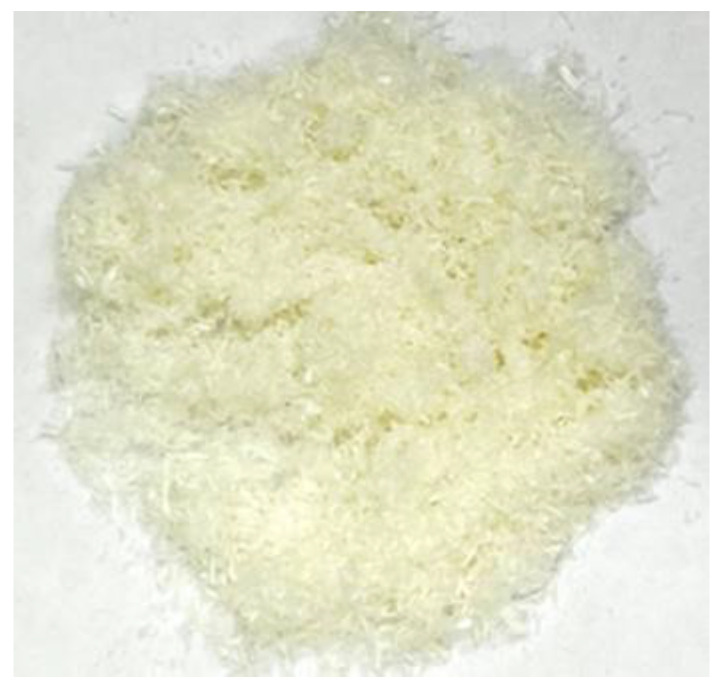
Cellulosic product obtained by peroxide delignification of spruce wood in the CH_3_COOH–H_2_O_2_–H_2_O medium over the sulfuric acid catalyst.

Delignification of spruce wood was carried out in a glass reactor in the form of a round-bottom flask with a mechanical stirrer and a reflux condenser. The delignifying reaction mixture contained chemically pure acetic acid (25 wt.%) (CHRS, Ufa, Russia), hydrogen peroxide (6 wt.%) (CHRS, Ufa, Russia), and distilled water. The liquor-to-wood ratio was 15. Delignification proceeded at a temperature of 100 °C for 3 h under constant stirring. The process was catalyzed by H_2_SO_4_ (98 wt.%) (CHRS, Ufa, Russia) added in an amount of 1 wt.% of the wood weight. The cellulosic product was separated from the reaction solution by filtration, washed with distilled water until the neutral reaction of wash water, and dried at 105 °C to constant weight. The cellulose product was a powder consisting of white needle fibers (Figure 1).

The chemical composition of the cellulosic product included, wt.%: cellulose—92.7, lignin—1.3, and hemicellulose—5.4. The degree of polymerization (DP) is 650 and the crystallinity index (IC) is 0.69.

### 2.2. Obtaining Microcrystalline and Microfibrillated Cellulose

**Figure 2:**
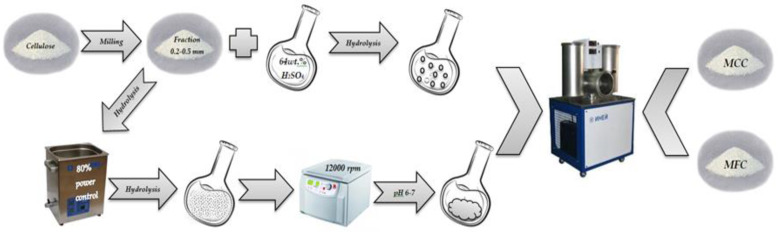
Scheme of the process obtained of the MCC and MFC.

Cellulose was preliminarily ground in a MUL m laboratory mill to obtain particles of 0.2–0.5 mm. MCC was obtained by the room temperature (25 °C) hydrolysis, for which a sample of cellulose (1.0 g) was mixed with 25 mL of a 64 wt.% H_2_SO_4_ solution. The reaction time ranged from 30 to 90 min. MFC was obtained under the same conditions by the sulfuric acid hydrolysis of cellulose in an ultrasonic bath (Grad, Grad technology, Moscow, Russia) 35 kHz; 55 W under the 80 % power control. The process temperature was kept at 25 °C by adding ice to the ultrasonic bath. The hydrolysis reaction was stopped by adding a tenfold volume of cold distilled water (∼10 °C). The obtained suspensions were centrifuged at a speed of 12,000 rpm in 4–5 cycles (OHAUS Multi–Pro FC5718, OHAUS, Greifensee, Switzerland) for 15 min until pH 6–7. MCC and MFC samples were frozen at –18 °C for 48 h and brought to constant weight by freeze drying (Iney-6, IBI RAS, Pushchino, Russia). The process is schematically illustrated in Figure 2.

The MCC and MFC yields were calculated gravimetrically.

### 2.3. Analysis of the Degree of Polymerization

The DP values for MCC and MFC samples were determined from the specific viscosity of their solutions in the copper-ammonia complex with a capillary viscometer (VPZh-3, Russia).

### 2.4. Elemental Analysis

The elemental composition of the cellulose samples was determined on a element analyzer (Vario El Cube, Elementar, Langenselbold, Germany) upon controlled combustion in pure oxygen at a constant furnace temperature of up to 1200 °C with the adsorption separation of the combustion products and their detection by the thermal conductivity.

### 2.5. Fourier-Transform Infra-Red Spectroscopy

The Fourier-transform infrared (FTIR) spectra were recorded on a FTIR spectrometer (Bruker Tensor 27, Bruker, Heidelberg, Germany) in the region of 4000–400 cm^−1^ and processed in the OPUS software package, version 5.0. The samples for the FTIR study were pressed in tablets containing 4 mg of the product in a potassium bromide matrix.

### 2.6. X-ray Diffraction Analysis

X-ray diffraction (XRD) patterns of the MCC and MFC samples were obtained on a diffractometer (PANalytical X’Pert Pro, Malvern Instruments Ltd., Malvern, UK), CuKα radiation (λ = 0.154 nm) in a cuvette 2.5 cm in diameter in the 2θ angle range from 10 to 50° with a step of 0.01°. The CI values were calculated using the Segal formula [24]. The crystallite size was determined by the Scherrer equation [25].

### 2.7. Scanning Electron Microscopy

The surface morphology of the cellulose samples was studied on a scanning electron microscope (SEM) (Hitachi TM-4000, Hitachi High-Tech Corporation, Tokyo, Japan) with a SwiftED3000 energy dispersive attachment (Oxford Instruments Analytical Ltd., Oxford, UK) at an accelerating voltage of 15 kV and a resolution of 20 μm.

### 2.8. Dynamic Light Scattering

The hydrodynamic diameter of MCC and MFC particles was measured by the dynamic light scattering (DLS) method using a spectrometer (Zetasizer Nano ZS, Malvern Instruments Ltd., Malvern, UK), which was also used to measure the ζ-potentials of suspended particles. The measurements were performed in polycarbonate cuvettes with Pd electrodes at a temperature of 20 °C from the electrophoretic mobility without adding a supporting electrolyte or adjusting pH.

### 2.9. Thermogravimetric Analysis

The thermogravimetric analysis (TGA) was carried out on a synchronous thermal analyzer (NETZSCH STA 449 F1 Jupiter instrument, Netzsch, Selb, Germany). The cellulose samples were analyzed in the argon atmosphere upon heating from 30 to 900 °C at a rate of 10 °C/min^−1^. The measured data were processed in the NETZSCH. Proteus Thermal Analysis. 5.1.0 software.

### 2.10. Water Holding Capacity Analysis

To determine the water holding capacity (WHC) of the initial cellulose, the weighed MCC and MFC samples (0.5 g) were added to distilled water (20 mL) and thoroughly mixed. After soaking, the samples were kept for 24 h and then the suspension was centrifuged at a speed of 4000 rpm for 10 min. The centrifugate was decanted and the wet pulp samples were weighed. The WHC values were calculated as the amount of water retained by the sample using the equation [26]:

$$\mathrm{WHC}=\left(\frac{m_{2}-m_{1}}{m_{1}}\right),$$

where m_2_ is the weight of the wet pulp sample, g; m_1_ is the initial weight of the sample, g.

### 2.11. DPPH Radical Scavenging Assay

The antiradical activity of the MCC and MFC was determined using the method described in [27,28]. To perform the UV measurements, a fresh solution of 1,1-diphenyl-2-picrylhydrazyl (DPPH) (TCI, Tokyo, Japan) in ethanol (0.2 mmol/L) was prepared. The MCC and MFC samples were also dissolved in ethanol in concentrations of 0.5, 2, and 5 mg/mL. The test solutions contained a cellulose solution (1 mL) thoroughly mixed with 2 mL of DPPH and 2 mL of ethanol. The mixtures were incubated at room temperature for 30 min in the dark. After that, the absorbance was measured at 517 nm against a blank. In this study, vitamin C (Vc) (LLC Ozon, Zhigulevsk, Russia) was used as a positive control.

The absorbance of DPPH radical was calculated as:

$$\mathrm{DPPH}\;\mathrm{Radical}\;\mathrm{Scavenging}\;\mathrm{Ability}\;(\%)=\left(1-\frac{A_{S}-A_{B}}{A_{C}}\right)*100\%,$$

where A_S_ is the absorbance of the test sample mixed with the DPPH solution, A_B_ is the absorbance of the sample without DPPH solution, and A_C_ is the absorbance of the DPPH solution without a sample.

## 3. Results

The main objective of acid hydrolysis is to remove the amorphous areas from cellu-lose microfibrils, which leads to a decrease in the DP and an increase in the CI, while the ultrasonic treatment promotes the destruction of microfibrils with the formation of na-nofibers [29,30]. The crystallinity and size of nanofibrils depend mainly on the hydrolysis and ultrasonic treatment time [31]. Here, the DP of the cellulose product was reduced by using the sulfuric acid hydrolysis in the MCC synthesis and the H_2_SO_4_ hydrolysis combined with the ultrasonic treatment in the MFC synthesis.

### 3.1. Degree of Polymerization and Yield of the MCC and MFC

**Figure 3:**
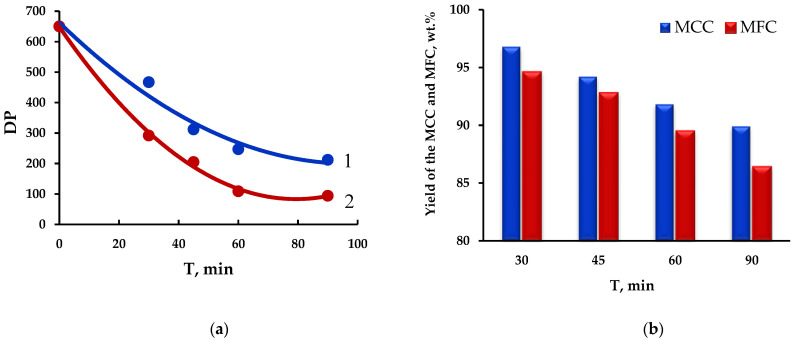
Effect of hydrolysis time: (**a**) on the change in DP 1–MCC; 2–MFC and (**b**) yield of MCC and MFC.

Figure 3 illustrates the effect of the hydrolysis time on the DP and MCC and MFC yields.

With an increase in the time of hydrolysis under ultrasonic exposure from 30 to 90 min, the MFC yield decreased stronger (86.4 wt.%) than the MCC yield (89.9 wt.%) did for the same time (Figure 3b).

During the hydrolysis process, the ultrasonic irradiation removes residual hemicel-luloses and, as was shown in [32], the partially crystalline cellulose areas are hydrolyzed along with the amorphous ones. In this case, the DP decreases to 94 for MFC and to 212 for MCC for the same hydrolysis time (90 min).

**Figure 4:**
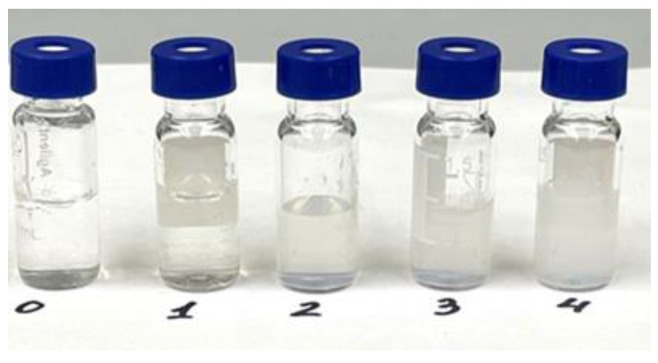
Photographs of the MFC suspension with nanofiber contents of (**0**) 0.5, (**1**) 1.0, (**2**) 1.5, (**3**) 2.0, and (**4**) 3.0 mg/L.

To demonstrate the stability, the MFC colloidal suspensions were additionally tested at different nanofiber concentrations in the aqueous solution for 20 days (Figure 4). As can be seen in Figure 4, neither separation nor sedimentation was observed.

**Figure 5:**
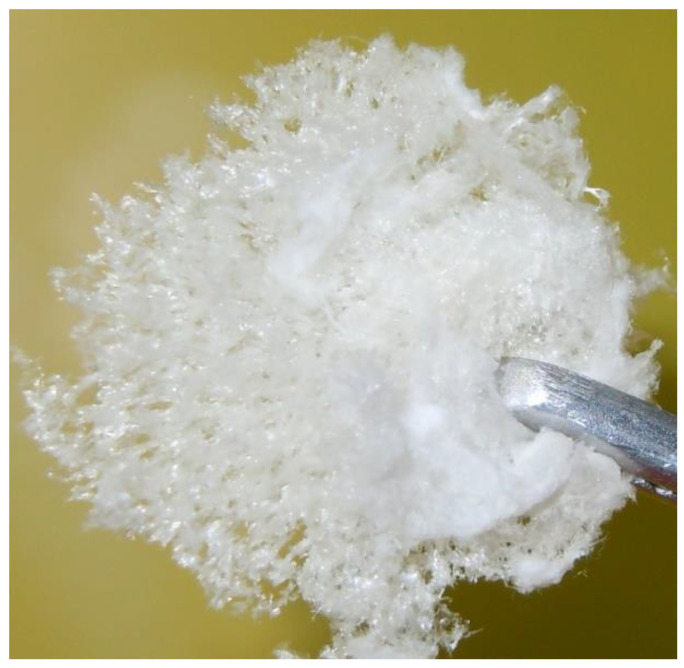
Photo of the obtaining MFC after freeze drying.

In this study, the obtained highly concentrated suspensions were dried in a low-temperature freeze dryer after preliminary freezing at −18 °C, which does not facilitate the formation of a film material under these conditions. The obtained MFC is shown in Figure 5.

The initial cellulose and obtained MCC and MFC samples were characterized by elemental analysis, FTIR spectroscopy, XRD and TGA, DLS, SEM, and estimation of the antiradical activity.

### 3.2. Elemental Analysis of the MCC and MFC

The data of the elemental analysis of the MCC and MFC samples (Table 1) show that the sulfur content increases by a factor of more than 5, which unambiguously indicates the formation of a sulfated cellulosic product. In addition, the calculated O/C atomic ratio is 0.91, which is higher than the theoretical value (0.83) based on C_6_O_5_ for pure cellulose.

### 3.3. FTIR Spectroscopy Analysis of the MCC and MFC

**Figure 6:**
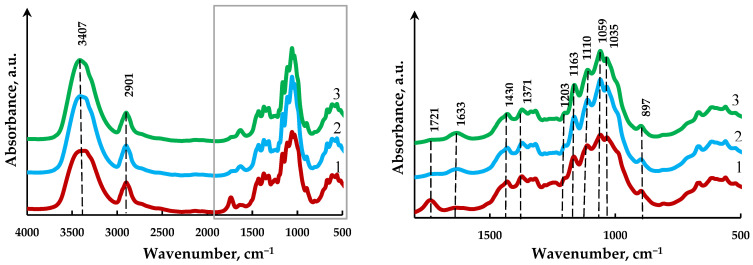
IR spectra of cellulose samples: (**1**) original cellulose; (**2**) MCC; (**3**) MFC.

The recorded IR spectra of the cellulose samples make it possible to identify free functional groups by absorption bands and evaluate a change in the supramolecular structure. Figure 6 presents the IR spectra of the initial cellulose and the MCC and MFC samples. The spectra of the cellulose samples are almost identical, which speaks about the similarity of their structures typical of form-I cellulose with characteristic absorption bands in three frequency ranges. The strong absorption of the OH groups linked by hydrogen bonds at 3407 cm^−1^, –CH, and –CH_2_ stretching vibrations at 2901 cm^−1^, and C=O and C–C absorption bands in the range of 1200–900 cm^−1^ are observed [33].

The absorption bands at 1163 cm^−1^ correspond to the stretching vibrations of C–O–C bonds of the glycosidic ring and the absorption band at 1203 cm^−1^ suggests the existence of sulfate groups on the cellulose surface [34]. The absorption bands at 1430 и 1371 cm^−1^ correspond to the bending vibrations of C–OH bonds.

The band at 1430 cm^−1^ is related to the crystalline cellulose area and the peak at 897 cm^−1^ belongs to the amorphous one [35]. In all cellulose samples, the primary hydroxyl group appears as an absorption band at 1033 cm^−1^, while the secondary hydroxyl group absorbs at 1059 and 1110 cm^−1^ [33]. The absorption band at 1633 cm^−1^ is related to adsorbed water and can be observed for all the samples. The absence of an absorption band at 1721 cm^−1^ corresponding to and being responsible for vibrations of ester bonds and acetyl groups of hemicellulose in the MCC and MFC spectra (2 and 3 in Figure 6) is evidence for their removal during the hydrolysis and ultrasonic treatment [36].

### 3.4. X-ray Diffraction Analysis of the MCC and MFC

**Figure 7:**
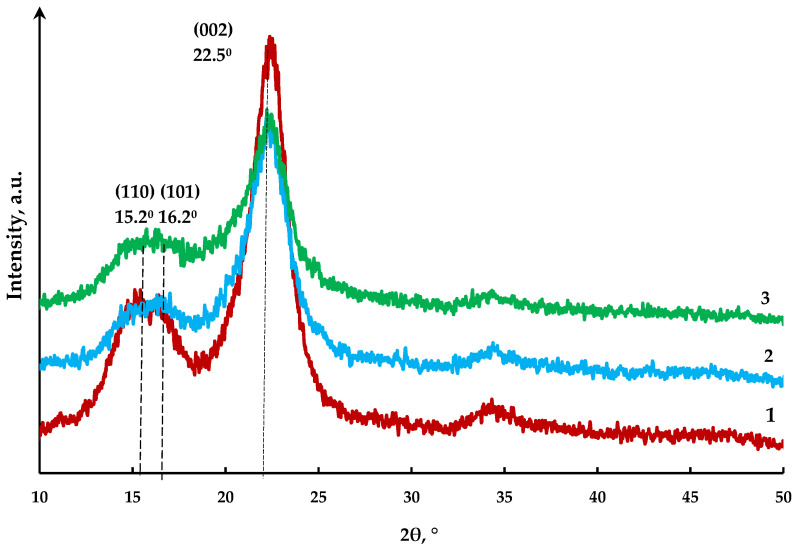
X-ray patterns of cellulose samples: (**1**) original cellulose; (**2**) MCC; (**3**) MFC.

The CI values of the cellulose samples were estimated by the XRD analysis. The XRD patterns of the initial cellulose, MCC, and MFC are presented in Figure 7.

The XRD study revealed two peaks in the 2θ angles range of 15.2–16.2° and a sharp peak at 22.5°, which are typical of cellulose [37]. The presence of these peaks for all samples suggests that the CI of cellulose changed neither during the hydrolysis nor during hydrolysis combined with the ultrasonic treatment. Hydrolysis of the initial cellulose facilitates the removal of amorphous areas, which is accompanied by a decrease in the intensity of the maxima in the 2θ angles range of 15.2–16.2° in the XRD patterns of the MCC and MFC samples (curves 2, 3 in Figure 7). Cellulose obtained by peroxide delignification of spruce wood is characterized by a CI value of 0.69. The acid hydrolysis of cellulose and co-hydrolysis with the ultrasonic treatment lead to the breaking of polymer chains in the residual amorphous areas of cellulose, which is accompanied by an increase in the CI to 0.76 and 0.74 for MCC and MFC, respectively. This result makes a contrast to the data presented in [38], where it was shown that the crystalline areas in nanofibers are damaged under the ultrasound exposure, which decreases the CI. On the other hand, the authors of [39] demonstrated that the ultrasonic treatment of cellulose reduces the CI of nanocelluloses insignificantly.

### 3.5. Scanning Electron Microscopy of the MCC and MFC

**Figure 8:**
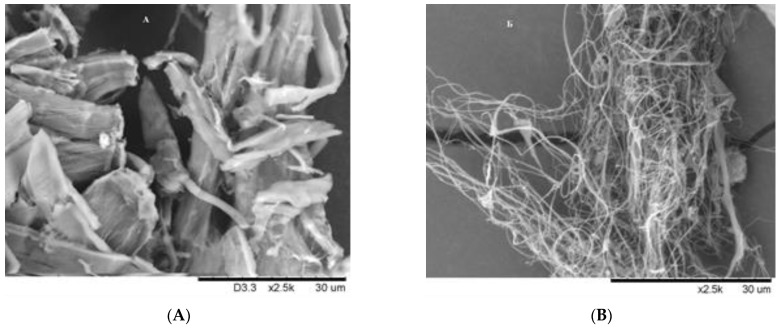
Micrographs of cellulose samples: (**A**) MCC; (**B**) MFC.

The acid hydrolysis and ultrasonic treatment of cellulose promote the removal of hemicelluloses, destruction of amorphous areas in microfibrils, and transverse splitting of cellulose fibers by means of breaking of interfibrillar hydrogen bonds along the cellulose longitudinal axis with the formation of nanofibers (see the electron microscopy images in Figure 8).

During the sulfuric acid hydrolysis, microcrystalline cellulose, consisting of a set of different sizes is formed (Figure 8A). The MFC samples obtained by hydrolysis combined with the ultrasonic treatment represent a network of smooth fibrils with a fairly uniform particle size (Figure 8B).

### 3.6. Dynamic Light Scattering Analysis of the MCC and MFC

**Figure 9:**
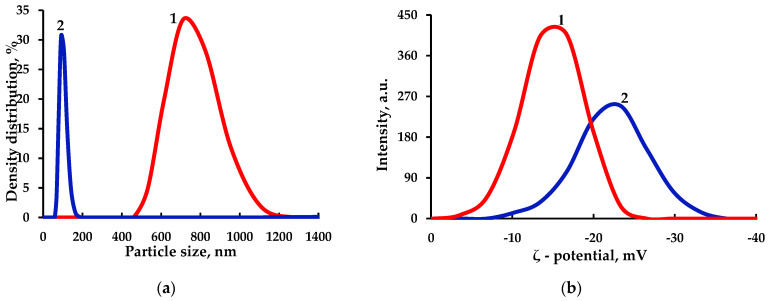
Hydrodynamic particle diameter (**a**) and ζ-potential of suspended particles (**b**) of nanocellulose samples 1–MCC and 2–MFC.

To establish the fiber size distribution in the 0.01% MCC and MFC aqueous suspensions, the hydrodynamic particle diameter was measured by DLS and the ζ-potential of suspended particles was found from the electrophoretic mobility (Figure 9).

The MFC particle size distribution is narrow and the average nanofiber size is 91.2 nm (curve 1 in Figure 9a), while for MCC a wide range (from 400 to 1200 nm) was obtained (curve 2 in Figure 9a). The MCC average particle size is 712.3 nm.

The ζ-potential is an important parameter for studying the stability of dispersion of cellulose fibers in an aqueous suspension. In this work, the average ζ-potential was found to be −13.4 mV for the MCC aqueous suspension (curve 2 in Figure 9b) and −23.3 mV for the MFC aqueous suspension (curve 1 in Figure 9b). The negative ζ-potential value is explained by the presence of negatively charged sulfate groups on the nanoparticles surface and points out the high stability of the MFC suspensions, since the obtained value is below −15 mV, which is the minimum value for the onset of particle agglomeration and separation of suspensions [40].

### 3.7. Thermogravimetric Analysis of the MCC and MFC

**Figure 10:**
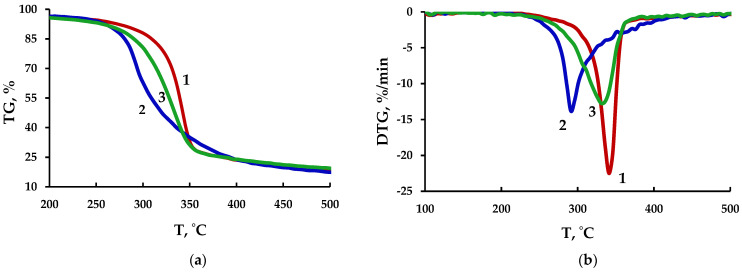
Curves (**a**) of TG and (**b**) DTG of cellulose samples: 1–initial cellulose; 2–MCC; 3–MFC.

Study of the thermal properties of MCC and MFC is of great importance for their use as reinforcing fillers for biocomposites. Figure 10 shows thermogravimetric (TG) and differential thermogravimetry (DTG) curves for initial cellulose and MCC and MFC samples.

According to the presented data, the thermal decomposition of all the samples occurs in two stages. The thermal destruction of cellulose includes, as a rule, dehydration, depolymerization, and splitting of glycosidic units with the formation of a charred residue. The first weight loss in all samples is observed in the temperature range of 45–120 °C at almost the same rate of 0.59–0.64%/min and amounts to about 6 wt.%, which corresponds to evaporation of freely bound moisture from the surface [41]. The second stage of the thermal destruction of the initial cellulose begins at 270 °C and attains its maximum at 342.8 °C (curve 1 in Figure 10b), while the maximum rate for MCC is observed at 292.8 °C, and the decomposition rate attains its maximum at 360 °C (curve 2 in Figure 10b). The intense decomposition ends at a temperature of 360 °C for the initial cellulose and at 410 °C for the MCC sample. The thermal decomposition of MFC starts later, at 285 °C, and attains its maximum at 333.9 °C (curve 3 in Figure 10b). The weight loss in the second stage of the thermal decomposition is 78.9, 81.1, and 77.5 wt.% for the initial cellulose, MCC, and MFC, respectively.

The lower thermal stability and wider decomposition temperature range for MCC as compared with the initial cellulose is related to the formation of particles in a wide size range. The thermal stability of MFC decreases due to the formation of nanofibers with a larger surface area and partial disruption of the crystalline structure of cellulose during the acidic hydrolysis with the ultrasonic treatment [42,43]. In this case, during the acidic hydrolysis of cellulose, hydroxyl groups are replaced by sulfate ones on the MCC and MFC particle surface, which also reduces the thermal stability [41,44,45].

### 3.8. Textural Characteristics and Water Holding Capacity Analysis of the MCC and MFC

The porous structure of the MCC and MFC samples was characterized using the specific surface area (S_BET_), total pore volume (V_pore_), and average pore size (Table 2).

The hydrolysis combined with the ultrasonic treatment increases the specific surface of the MFC sample by almost 2.5 and the average pore size by a factor of 1.5 as compared with the obtained sulfuric acid MCC sample; the total pore volume does not significantly increase.

A decrease in the particle size and an increase in the specific surface area and pore size of MFC leads to the growth of its WHC. The WHC of MCC (4.0 g/g) after the sulfuric acid hydrolysis and the WHC of MFC (5.3 g/g) after the hydrolysis with the ultrasonic treatment increased by a factor of 1.5–2.0 as compared with the value for the initial cellulose (2.7 g/g), which is consistent with the literature data [46,47].

### 3.9. Scavenging Activity of the DPPH Radicals

The method based on the free radical scavenging modeling allows one to determine the antiradical activity of potential antioxidants, including polysaccharides. The DPPH solution often used as an analog of free radicals demonstrates the strong absorption at 517 nm [27,28]. The antioxidant can bind to one DPPH electron with the formation of a stable yellow compound diphenylpicrylhydrazine with the absorption decreasing with an increase in the antioxidant concentration.

**Figure 11:**
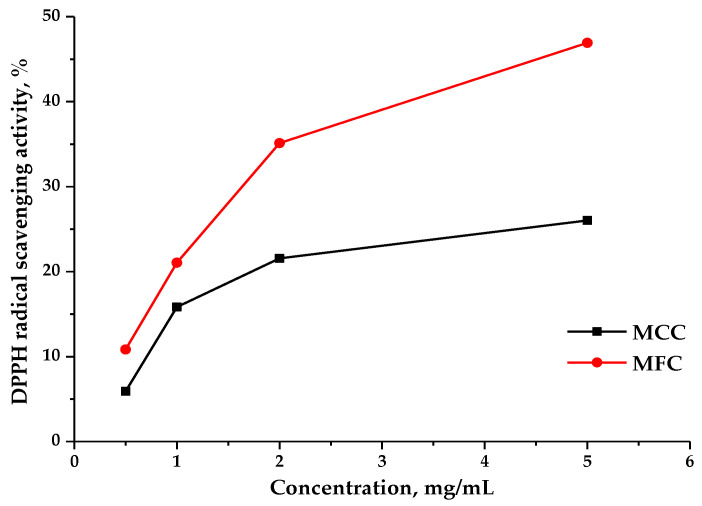
DPPH radical scavenging at different concentrations of the MCC and MFC in solution.

The absorption capacities of the MCC and MFC on the DPPH radical were determined and the results are shown in Figure 11.

In this study, it was shown that MCC and MFC have a pronounced inhibitory effect of DPPH radicals; the higher the concentration of the MCC and MFC samples in the solution, the higher the free radical inhibition level. However, as can be seen in Figure 11, the MFC sample has a stronger DPPH radical scavenging effect over the entire concentration range (up to 46.9%), in contrast to the MFC sample, which demonstrated a free radical scavenging capacity of up to 26.1% at all the concentrations tested.

## 4. Conclusions

In this study, a new approach to obtaining microfibrillated cellulose with a low DP by the sulfuric acid hydrolysis with simultaneous ultrasonic treatment under mild conditions (temperature 25 °C, power control 80%). For comparison, the sulfuric acid hydrolysis of cellulose obtained by oxidative delignification of spruce wood was carried out under the same conditions.

The composition and properties of the initial cellulose, MCC, and MFC were characterized by the chemical and physicochemical methods.

It is shown that during the hydrolysis with the ultrasonic treatment for 90 min, the MFC yield was 86.4 wt.% at the low DP (94). During the sulfuric acid hydrolysis without ultrasonic treatment, the MCC yield was 89.9 wt.%, at a DP of 212.

The sulfur content in the MCC and MFC samples increases by a factor of more than 5 (from 0.12 to 0.52 and 0.66 wt.%, respectively), which unambiguously indicates the formation of a sulfated cellulosic product.

It was found that the obtained MCC and MFC samples retain the structure of cellulose I and have ICs of 0.76 and 0.74, respectively.

It was noted that the FTIR spectra of the initial cellulose and the MCC and MFC samples differ insignificantly, which evidences for the similarity of their structures; however, the absence of an absorption band at 1721 cm^−1^ in the MCC and MFC spectra suggests the removal of ester bonds and acetyl groups of hemicellulose during the hydrolysis and ultrasonic treatment.

It was shown that MFC particles represent a network of fibrils with an average size of 91.2 nm. A wide size range (from 400 to 1200 nm) for MCC was found. The average MCC particle size was found to be 712.3 nm. The ζ-potential of the MFC aqueous suspension (−3.3 mV) is indicative of its high stability.

It was noted that MFC has the high thermal stability with the maximum decomposition temperature of 333.9 °C. The specific surface of MFC is greater than that of MCC by a factor of 2.5, which ensures the higher WHC.

It was found that MFC has the more pronounced DPPH radical scavenging effect at all the investigated concentrations (up to 46.9%). The MCC sample exhibited a weaker effect on the removal of DPPH radicals (26.1%) over the entire concentration range.

Thus, the hydrolysis combined with the ultrasonic treatment for the isolation of low-DP MFC from spruce wood pulp makes it possible to decrease the number of stages, reduce energy costs, and expand the range of application.

## Figures and Tables

**Table 1:** Elemental composition of the initial cellulose and MCC and MFC samples.

Sample	Elemental Composition, wt.%				H/C Atomic Ratio	O/C Atomic Ratio
	C	H	S	O (diff)		
Initial cellulose	42.6	6.5	0.12	50.78	1.83	0.85
MCC	41.2	6.8	0.52	51.48	1.93	0.91
MFC	41.0	6.9	0.66	51.44	1.95	0.91

**Table 2:** Textural characteristics of the MCC and MFC.

Samples	S_BET_, m^2^/g	V_pore_., cm^3^/g	Average Pore Size, nm
MCC	5	0.009	6.8
MFC	12	0.010	11.4

## Data Availability

All data generated during this study are included in the article.
